# Immune-Enhancing Effects of Crude Polysaccharides from Korean Ginseng Berries on Spleens of Mice with Cyclophosphamide-Induced Immunosuppression

**DOI:** 10.4014/jmb.2110.10021

**Published:** 2021-12-17

**Authors:** Ju Hyun Nam, JeongUn Choi, Chaiwat Monmai, Weerawan Rod-in, A-yeong Jang, SangGuan You, Woo Jung Park

**Affiliations:** 1 Department of Wellness-Bio Industry, Gangneung-Wonju National University, Gangneung 25457, Republic of Korea; 2 Department of Marine Food Science and Technology, Gangneung-Wonju National University, Gangneung 25457, Republic of Korea

**Keywords:** Korean ginseng berry, polysaccharides, cyclophosphamide, immune-enhancing effect

## Abstract

*Panax ginseng* C. A. Meyer is well known as traditional herbal medicine, and ginseng berries are known to exhibit potential immune-enhancing functions. However, little is known about the in vivo immunomodulatory activity of Korean ginseng berries. In this study, crude Korean ginseng berries polysaccharides (GBP) were isolated and their immunomodulatory activities were investigated using cyclophosphamide (CY)-induced immunosuppressive BALB/c mice. In CY-treated mice, oral administration of GBP (50-500 mg/kg BW) remarkably increased their spleen sizes and spleen indices and activated NK cell activities. GBP also resulted in the proliferation of splenic lymphocytes (coordinating with ConA: plant mitogen which is known to stimulate T-cell or LPS: endotoxin which binds receptor complex in B cells to promote the secretion of pro-inflammatory cytokines) in a dose-dependent manner. In addition, GBP significantly stimulated mRNA expression levels of immune-associated genes including interleukin-1β (*IL-1β*), *IL-2*, *IL-4*, *IL-6*, tumor necrosis factor-α (*TNF-α*), interferon-γ (*IFN-γ*), toll-like receptor 4 (*TLR-4*), and cyclooxygenase-2 (*COX-2*) in CY-treated mice. These results indicate that GBP is involved in immune effects against CY-induced immunosuppression. Thus, GBP could be developed as an immunomodulation agent for medicinal or functional food application.

## Introduction

The immune system is an organization of cells and molecules that defends the body against infection. It consists of two main subsystems: the innate immune system and the adaptive immune system [1]. Immune responses such as immune organ indices (bone marrow, spleen, thymus) and changes in the population of immune cells (macrophage, splenocytes, neutrophils, natural killer cells) are essential mechanisms for enhancing the body’s immune function [2]. These responses activate a variety of immunomodulating functions by producing various cytokines (such as interferon-γ (IFN-γ), tumor necrosis factor-α (TNF-α), interleukin-2 (IL-2), and IL-6) and other inflammatory mediators [3, 4]. Natural products from plant extracts have the ability to stimulate the host immune system and can serve as potential therapies to substitute for chemotherapy [5-7].

Anti-cancer drugs such as cyclophosphamide (CY) have been used in chemotherapy to treat cancer, autoimmune diseases, and immune-mediated diseases. However, they can produce serious side effects such as cytotoxicity and oxidative stress [8]. In addition, they can damage the DNAs of normal cells and lead to a significant immunosuppressive condition [5, 9]. In such case, it is important to recover the immunosuppressive condition [8]. The CY-induced immunosuppressive mice model has been used in many previous studies to assess the immunomodulatory effects of functional materials [10-12].

Polysaccharides are a class of bioactive macromolecular substances with beneficial pharmacological activities that include anti-tumor, antioxidant, anti-diabetic, and immunomodulatory effects [13-15]. Polysaccharides extracted from plants such as *Lonicera japonica*, *Codonopsis pilosula*, *Sargassum fusiforme*, *Panax quinquefolius* L., and *Lycium ruthenicum* have been reported to exert immunomodulatory effects in CY-induced mice [5, 12, 16-18]. *Panax ginseng* C. A. Meyer is one of the most commonly used traditional medicinal plants in Korea, China, and some other East Asian countries [19]. Ginseng berry has been reported to exhibit immune-modulating, anticancer, anti-aging, and anti-diabetic effects [20-24]. Polysaccharides are major bioactive constituents isolated from *P. ginseng* [25, 26]. It has been reported that polysaccharides of *P. ginseng* can exert immunomodulatory activities in both in vitro and in vivo systems [27-29]. Crude polysaccharides from *P. quinquefolius* can increase macrophage phagocytosis and nitric oxide (NO) production as well as splenic lymphocyte proliferation [17]. Polysaccharides from Korean ginseng after cellulase- and α-amylase-based extraction can enhance spleen and thymus indices, lymphocyte proliferation, leukocyte count, NK cell activity, and serum levels of *IL-2*, *IL-6*, and *IFN-γ* in CY-induced immunosuppressed mice [30]. Moreover, we recently reported that Korean ginseng berries show immune-enhancing effects in RAW 264.7 cells [31]. However, still little is known about the immunomodulatory effect of polysaccharides from Korean ginseng berry in CY-induced immunosuppressive mice model. Therefore, our objective in the current study was to investigate the in vivo immune-enhancing properties of crude Korean ginseng berry polysaccharides (GBP).

## Materials and Methods

### Animals

Six-week-old male BALB/c mice (21-23 g) were obtained from the Central Lab, Animal Inc. (Korea). Animals were housed under standard conditions with temperature maintained at 22 ± 2°C and a 12-h/12-h dark/light cycle for at least one week before the experiments. A standard laboratory diet and water were provided to all mice. All experimental processes were approved by the Gangneung-Wonju National University Committee.

### Korean Ginseng Berry Polysaccharides

Korean ginseng berries were obtained from Boeun, Chungcheong Province, Korea. Korean GBPs with molecular weights of 328.4 and 54.2 × 10^3^ (g/mol) comprising rhamnose (4.0%), arabinose (19.8%), mannose (2.2%), glucose (27.7%), and galactose (46.3%) were prepared as described in our previous report [31].

### Immunosuppressive Treatments

In this study, 40 mice were randomly divided into 8 groups (*n* = 5 per group) for various treatments (Table 1). CY was used to induce immunosuppressive models by intraperitoneal (IP) injection at a dose of 80 mg/kg body weight (BW). Levamisole (LEV) and commercial red ginseng syrup were used as positive controls at doses of 40 mg/kg BW and 100 mg/kg BW, respectively. LEV is an immunomodulatory agent that can modulate cell-mediated, *i.e.*, T cell-directed immunity [3, 32]. Ginseng is a commercially available product that can modulate the immune system [33]. Mice were sacrificed at 24 h after the last treatment.

### Measurement of Spleen Index

The spleen was dissected and weighed. The spleen index was calculated according to the formula as follows:

Spleen index (mg/g) = the spleen weight/the body weight of mouse.

### Preparation of Mouse Splenocytes

Splenocytes were isolated from spleens of BALB/c mice. After weighing, spleens of mice were placed in ice-cold PBS for splenocyte isolation. These splenocytes were extracted using RBC Lysis Buffer (eBioscience, USA) according to the manufacturer’s instructions. Cells were adjusted to a concentration of 2 × 10^6^ cells/ml in RPMI-1640 medium (Gibco Laboratories, USA) supplemented with 1% fetal bovine serum and 1% streptomycin (100 μg/ml) penicillin (100 IU/ml) (Welgene, Korea).

### Measurement of Splenocyte Proliferation

EZ-Cytox Cell Viability Assay Kit (Daeillab Service, Korea) was used to evaluate splenic lymphocyte proliferation. Splenocytes were seeded into a 96-well plate and cultured at 37°C with 5% CO_2_ for 1 h. These cells were then stimulated with 5 μg/ml concanavalin A (Con A) as T cell mitogen or 10 μg/ml lipopolysaccharide (LPS) as B cell mitogen. After incubation at 37°C with 5% CO_2_ for 48 h, 25 μl EZ-Cytox reagent was added to each well and plates were incubated at 37°C with 5% CO_2_ for another 1 h. The absorbance at 450 nm was then measured using a microplate reader (EL800; BioTek, USA).

### Analysis of NK Cell-Mediated Cytotoxicity Assay

Splenocytes were co-cultured with or without YAC-1 cells (Korean Cell Line Bank) to obtain a 50:1 ratio of effector cells to NK-sensitive target cells. After 24 h incubation, the supernatant was collected after centrifugation at 250 ×g for 5 min. The activity of NK cells was assessed with a CytoTox 96 Non-Radioactive Cytotoxicity Assay Kit (Promega, USA). The absorbance at 490 nm was then measured.

### Quantitative RT-PCR (qRT-PCR) Analysis

Tri reagent (Molecular Research Center, Inc., USA) was employed to extract total RNAs from splenic lymphocytes. Total RNAs were then reverse-transcribed to cDNA using a High-Capacity cDNA Reverse Transcription Kit (Applied Biosystems, USA). To amplify the cDNA, the cDNA was subjected to qRT-PCR using TB Green Premix Ex Taq II (Takara Bio Inc., Japan) on a QuantStudio 3 Flex Real-Time PCR System (Thermo Fisher Scientific, USA). Amplification conditions were as follows: initial denaturation at 95°C for 30 s, followed by 40 cycles of 95°C for 5 s and 60°C for 34 s. Primer sequences are listed in Table 2.

### Statistical Analysis

The significance of differences was evaluated using one-way analysis of variance (ANOVA) followed by Tukey’s post-hoc test using Statistix 8.1 Statistics software (Statistix, USA). Significant difference between treatment groups was considered when the *p*-value was less than 0.05.

## Results

### Effect of GBP on Spleen Index in CY-Treated Mice

**Fig. 1:**
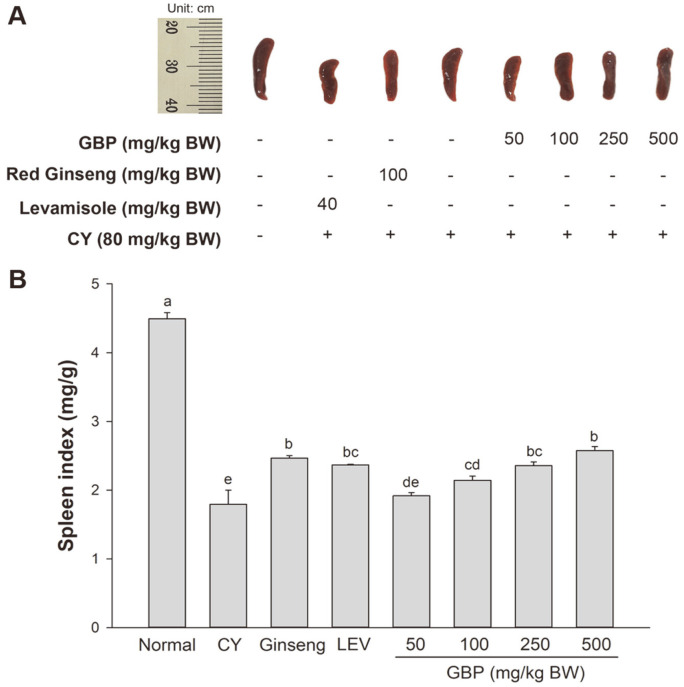
Effects of GBP at various concentrations on spleen size and spleen index. (**A**) Effects on spleen size, (**B**) Effects on spleen index. Results are expressed as means ± SD. Different letters (a-e) indicate significant (*p* < 0.05) differences between treatment groups.

The effects of ginseng berry polysaccharides (GBP) on spleen size and spleen indices of BALB/c mice are shown in Fig. 1. Compared with the normal group, the CY-treated group showed significant decreases of both spleen size and spleen index. Compared with the CY group, the LEV treatment group and the red ginseng syrup treatment group showed increased spleen index. GBP groups also showed significantly increased spleen indices in a dose-dependent manner (50, 100, 250, and 500 mg/kg BW).

### Effects of GBP on Splenic Lymphocyte Proliferation

**Fig. 2:**
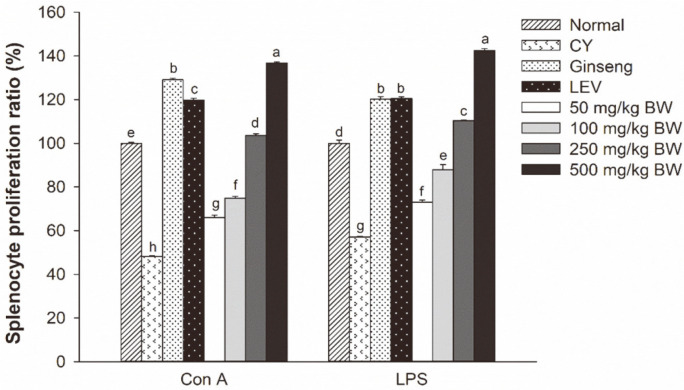
Effects of GBP on Con A- and LPS-induced splenocyte proliferation. Results are expressed as means ± SD. Different letters (a-h) indicate significant (*p* < 0.05) differences between treatment groups.

The effects of GBP on mitogen-stimulated spleen lymphocyte proliferation are shown in Fig. 2. The CY group showed significantly reduced Con A- or LPS-stimulated cell proliferation (48.1% and 57.1%, respectively) compared to the normal group. GBP at doses of 250 and 500 mg/kg BW significantly stimulated the proliferation of splenocytes induced by Con A or LPS compared to the CY group. The proliferation of lymphocytes of the group treated with GBP at 500 mg/kg BW was higher than those of positive control groups (red ginseng syrup and LEV).

### Effect of GBP on Splenic NK Cell Activity

**Fig. 3:**
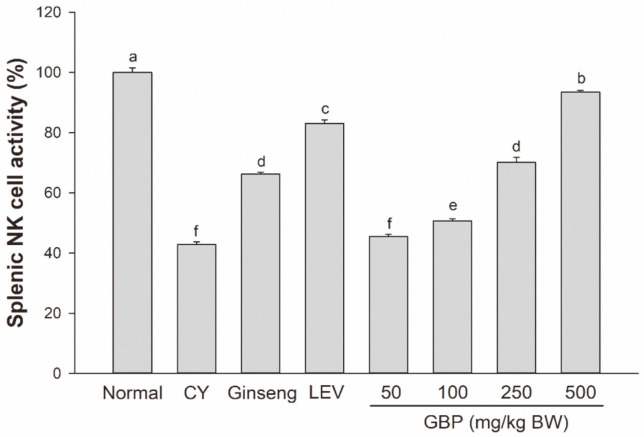
Effects of GBP on splenic NK cell activity. Results are expressed as means ± SD. Different letters (a-f) indicate significant (*p* < 0.05) differences between treatment groups.

As shown in Fig. 3, CY strongly inhibited the activity of NK cells to 42.9%. NK activity of the GBP group at 50 mg/kg BW showed little difference from that of the CY group. However, the NK cell activities of GBP treatment groups at doses of 100, 250, and 500 mg/kg BW were significantly higher than those of the CY group in a dose-dependent manner. In addition, GBP at a dose of 500 mg/kg BW recovered the splenic NK cell activity up to a level similar to that of the normal group.

### Effect of GBP on Gene Expression in CY-Treated Mice

**Fig. 4:**
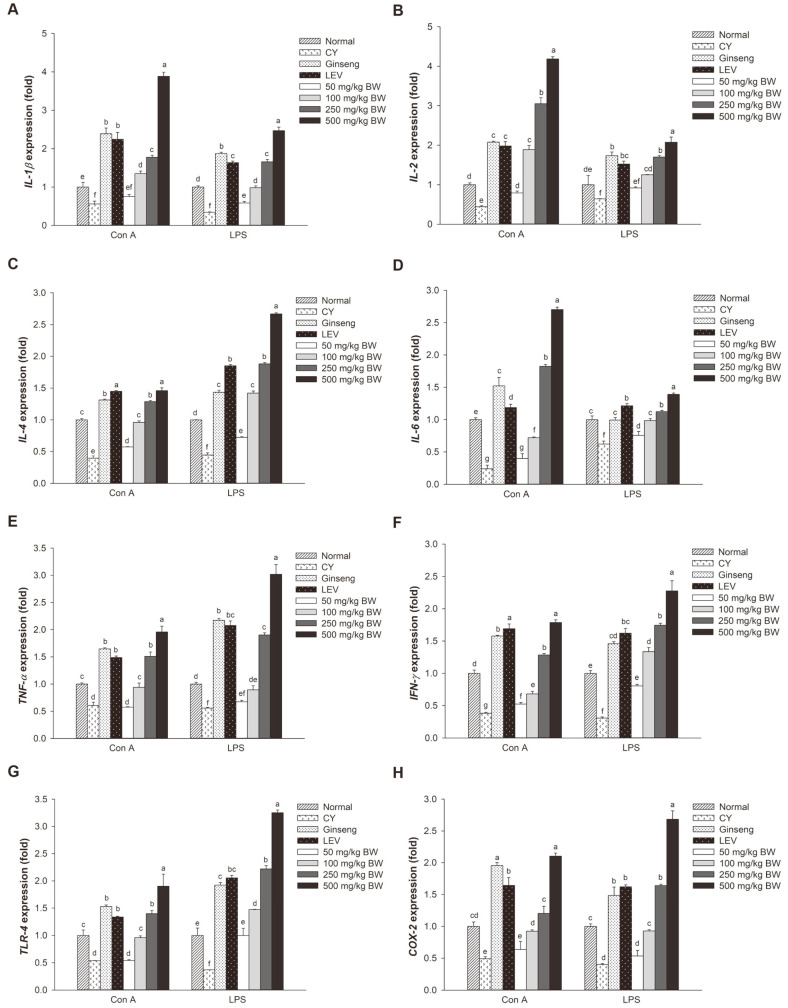
Effects of GBP on mRNA expression levels of immune-related genes in mitogen-stimulated splenic lymphocytes. (**A**) *IL-1β*, (**B**) *IL-2*, (**C**) *IL-4*, (**D**) *IL-6*, (**E**) *TNF-α*, (F) *IFN-γ*, (**G**) *TLR-4*, and (**H**) *COX-2* expression. Results are displayed as means ± SD. Different letters (a-g) indicate significant (*p* < 0.05) differences between treatment groups.

To assess whether GBP could stimulate immune responses in splenic lymphocytes stimulated by T-cell mitogen (Con A) and B-cell mitogen (LPS), real-time PCR was performed to examine mRNA expression levels of immune-related genes such as *IL-1β*, *IL-2*, *IL-4*, *IL-6*, *TNF-α*, *IFN-γ*, *TLR-4*, and *COX-2*. As shown in Fig. 4, mRNA expression levels of these genes in CY group were lower than those of the normal group. All GBP groups (50-500 mg/kg BW) showed markedly increased expression levels of immune-related genes than the CY group. GBP treatment groups, expression levels of *IL-1β*, *IL-2*, and *IL-6* were significantly upregulated by T-cell mitogens than by B-cell mitogens (Figs. 4A, 4B, and 4D). Conversely, *IL-4*, *TNF-α*, *IFN-γ*, *TLR-4*, and *COX-2* expression levels were significantly upregulated by B-cell mitogens than by T-cell mitogens (Figs. 4C, 4E-4H). At the highest doses (250 and 500 mg/kg BW), GBP also increased expression levels of these genes than the normal group.

## Discussion

In this study, we investigated the potential of Korean GBPs to activate immune systems in a CY-induced immunosuppressed mice model. Modulation of splenic lymphocytes was determined by cell proliferation, NK cell activity, and immune-associated gene expression. CY is an inducer of immunosuppression. It is a widely used alkylating agent for cancer therapy [8]. In mice models, the effect of CY could be shown as reduction of immune organ index. The capacity of the immune system decreases after CY treatment [5, 12]. In addition, immune-stimulating agents such as LEV and red ginseng syrup were applied as positive controls to recover the immunosuppressive condition induced by CY.

The spleen is one of the most critical immune organs. It plays an important role in the body’s immune function by regulating immune responses. Their weights and organ indices indicate the function of innate immunity to some extent [10, 34]. In this study, spleen indices of mice treated with LEV, red ginseng syrup, and GBP were higher than that of the CY group. Treatments with GBP (50-500 mg/kg BW) remarkably increased spleen weights of CY-treated mice, indicating that GBP could regulate immune organs to improve immune damage caused by CY (Fig. 1). Similar to our study results, Song *et al*. have reported that polysaccharides extracted from Korean ginseng with enzyme-assisted extraction can increase spleen and thymus indices, enhance the proliferation of lymphocytes and NK cell activity, and increase serum levels of *IL-2*, *IL-6*, and *IFN-γ* in CY-induced BALB/c mice [30].

Lymphocytes are major cellular components of the adaptive immune response. They can be divided mainly into T cells and B cells depending on their developmental site, surface, antigen, receptor, and function [3]. They are involved in activating the processes of antigen presentation and mitogen stimulation [35]. Splenic lymphocyte proliferation is an important biomarker for the activation of cellular and humoral immune responses [36]. This activity is induced by mitogen Con A or LPS, which is commonly used to evaluate T or B lymphocyte activity [34]. In the present study, proliferation assay results showed that GBP could significantly promote Con A- and LPS-stimulated splenocyte proliferation and restore its decrease induced by CY (Fig. 2). Especially, high doses of GBP (250-500 mg/kg BW) showed strong immune-enhancing effects on mitogen-induced proliferation. Similar to our results, American ginseng polysaccharides can also promote the proliferation of B cells or T cells in these dose ranges [17].

Wang *et al*. [24] have reported that ginseng fruit polysaccharides can inhibit tumor growth and lung metastasis, promote spleen lymphocyte proliferation, increase NK cell activities, and increase serum concentrations of IL- 2 and *IFN-γ* in a Lewis lung carcinoma (LLC)-bearing mouse model. Our results showed that NK cell activity was attenuated by CY administration, whereas GBP treatment significantly enhanced the activity of NK cells from splenocytes, similar to positive controls (red ginseng syrup and LEV).

After activation, splenic lymphocytes can secrete several cytokines, chemokines, and other inflammatory mediators to regulate functions of other innate and adaptive immune cells [16, 37]. Cytokines play an important role in immune responses by modulating immune-related cells (B cells, T cells, and NK cells) and non-immune cells (endothelial cells, epidermal cells, and fibroblasts) [3, 9]. Helper T cells (Th cells) are essential immunoregulatory cells of the body [38]. Th 1 cells can produce type 1 cytokines (IL-2, TNF-α, IFN-γ), while Th 2 cells can produce type 2 cytokines (IL-4, IL-5, IL-6, IL-10, IL-13) [39]. Many studies have shown that plant-derived polysaccharides can modulate the secretion of cytokines [4, 12, 16]. The present results showed that GPB treatment increased expression levels of cytokines (*IL-1β*, *IL-2*, *IL-4*, *IL-6*, *TNF-α*, and *IFN-γ*) in a dose-dependent manner and that high doses (250 and 500 mg/kg BW) of GBP could improve these cytokines more so than the normal control (Fig. 4). GBP treatment also upregulated *TLR-4* and *COX-2* expression depending on its concentration (Fig. 4). COX-2 is an inflammatory mediator. COX-2-mediated PGE_2_ plays an important role in IFN-γ and IL-12 production of BALB/c spleen cells [40]. *TLR-4* has a fundamental role in the initiation of adaptive immunity, activation of cells, and pathogen recognition. It can also induce the expression of *TNF-α*, *IL-1β*, and *IL-6* [4]. These results suggest that GBP could improve immune activity by upregulating the expression of cytokines induced by CY.

Korean GBPs exhibited potent immunomodulatory properties in a CY-induced immunosuppressed mice model. GBP treatment enhanced immune responses by improving the spleen index. It markedly restored splenocyte proliferation and NK cell activity. It also upregulated gene expression of immune-regulated cytokines. This is the first study to report that GBP possesses immune-enhancing activity in an in vivo mice model, suggesting that GBP could be developed as an effective immunomodulatory agent.

## Figures and Tables

**Table 1:** BALB/c mouse groups with different treatments.

Group	Treatment		

	Day 1 to 6 (orally)	Day 4 to 6 (IP)	Day 7 to 10 (orally)
Normal	Saline	-	Saline
CY	Saline	CY	Saline
Red ginseng	Ginseng syrup	CY	Ginseng syrup
LEV	Levamisole	CY	Levamisole
Tr. 1	GBP (50 mg/kg BW)	CY	GBP (50 mg/kg BW)
Tr. 2	GBP (100 mg/kg BW)	CY	GBP (100 mg/kg BW)
Tr. 3	GBP (250 mg/kg BW)	CY	GBP (250 mg/kg BW)
Tr. 4	GBP (500 mg/kg BW)	CY	GBP (500 mg/kg BW)

**Table 2:** List of primers used for real-time PCR analysis.

Gene	Accession No.	primer Sequence (5’ to 3’)
IL-1β	NM_008361.4	**Forward**: GGGCCTCAAAGGAAAGAATC **Reveres**: TACCAGTTGGGGAACTCTGC
IL-2	NM_008366.3	**Forward**: CCTGAGCAGGATGGAGAATTACA **Reveres**: TCCAGAACATGCCGCAGAG
IL-4	NM_021283.2	**Forward**: ACAGGAGAAGGGACGCCAT **Reveres**: GAAGCCCTACAGACGAGCTCA
IL-6	NM_031168.2	**Forward**: AGTTGCCTTCTTGGGACTGA **Reveres**: CAGAATTGCCATTGCACAAC
TNF-α	D84199.2	**Forward**: ATGAGCACAGAAAGCATGATC **Reveres**: TACAGGCTTGTCACTCGAATT
IFN-γ	NM_008337.3	**Forward**: CTCAAGTGGCATAGATGT **Reveres**: GAGATAATCTGGCTCTGCAGGATT
TLR-4	NM_021297.3	**Forward**: CGCTCTGGCATCATCTTCAT **Reveres**: GTTGCCGTTTCTTGTTCTTCC
COX-2	NM_011198.4	**Forward**: AGAAGGAAATGGCTGCAGAA **Reveres**: GCTCGGCTTCCAGTATTGAG
β-actin	NM_007393.5	**Forward**: CCACAGCTGAGAGGGAAATC **Reveres**: AAGGAAGGCTGGAAAAGAGC
